# The Combined Influence of Distance and Neighbourhood Deprivation on Emergency Department Attendance in a Large English Population: A Retrospective Database Study

**DOI:** 10.1371/journal.pone.0067943

**Published:** 2013-07-16

**Authors:** Gavin M. Rudge, Mohammed A. Mohammed, Sally C. Fillingham, Alan Girling, Khesh Sidhu, Andrew J. Stevens

**Affiliations:** 1 School of Health and Population Sciences, University of Birmingham, Birmingham, United Kingdom; 2 University of Bradford, Bradford, United Kingdom; 3 Welsh Health Specialised Services Team, Caerphilly, Wales, United Kingdom; University of Gävle, Sweden

## Abstract

The frequency of visits to Emergency Departments (ED) varies greatly between populations. This may reflect variation in patient behaviour, need, accessibility, and service configuration as well as the complex interactions between these factors. This study investigates the relationship between distance, socio-economic deprivation, and proximity to an alternative care setting (a Minor Injuries Unit (MIU)), with particular attention to the interaction between distance and deprivation. It is set in a population of approximately 5.4 million living in central England, which is highly heterogeneous in terms of ethnicity, socio-economics, and distance to hospital. The study data set captured 1,413,363 ED visits made by residents of the region to National Health Service (NHS) hospitals during the financial year 2007/8. Our units of analysis were small units of census geography having an average population of 1,545. Separate regression models were made for children and adults. For each additional kilometre of distance from a hospital, predicted child attendances fell by 2.2% (1.7%–2.6% p<0.001) and predicted adult attendances fell by 1.5% (1.2% –1.8%, p<0.001). Compared to the least deprived quintile, attendances in the most deprived quintile more than doubled for children (incident rate ratio (IRR)  = 2.19, (1.90–2.54, p<0.001)) and adults (IRR 2.26, (2.01–2.55, p<0.001)). Proximity of an MIU was significant and both adult and child attendances were greater in populations who lived further away from them, suggesting that MIUs may reduce ED demand. The interaction between distance and deprivation was significant. Attendance in deprived neighbourhoods reduces with distance to a greater degree than in less deprived ones for both adults and children. In conclusion, ED use is related to both deprivation and distance, but the effect of distance is modified by deprivation.

## Introduction

### Background

Presentation at Emergency Departments (EDs) whether by self referral or directed by other services, is an important route into acute hospital care.

The manner in which people interact with healthcare services, particularly EDs, has been shown to be strongly influenced by proximity in England [1], [2], [3], Scotland [4], Northern Ireland [5], Canada [6], the US [7], [8] and Sweden [9]. It has also been shown that higher degrees of socio-economic deprivation are associated with increased attendance in EDs [2], [3], [6].

### Importance

The provision of ED facilities is a high cost, high volume service. In England, it is estimated that there were over 19 million visits to EDs and Minor Injury Units (MIU) in 2007/08 [10]. In financial year 2008/09, the National Health Service estimated the cost of attendances at English EDs at over £1.3 billion [11]. Historically, hospitals with EDs were given fixed sums of money each year to provide the service by the local budget holders who financed the health needs of their populations, regardless of the actual demands on the service. Since 2006, ED attendances have come under the England's ‘Payment by Results’ (PbR) system [12] in which they are individually billed to the budget holder on a fee for service basis. Given the costs involved in providing the service, there has been considerable scrutiny of the extent to which EDs are being inefficiently or “inappropriately” used for less urgent needs instead of making an appointment with the General Practitioner (GP) that the patient is registered with. Also the ability of EDs to process patients quickly is seen as an indicator of performance, particularly in England where specific targets relating to ED waiting times have been centrally set by Government [13]. For these reasons it is important that those who plan, fund or manage EDs understand the factors that affect the demand for this service.

### Goals of this investigation

The study has three objectives. 1) To explore the effects of distance and deprivation on ED attendance using a larger and more heterogeneous population than has been studied before. 2) To determine if the relationship between distance and ED attendance varies by deprivation. 3) To see if proximity to Minor Injury Units (MIU) affects the extent to which populations use Emergency Departments in acute hospitals.

## Materials and Methods

### Study Design

We obtained counts of attenders at EDs for one financial year (1^st^ April 2007 to 31^st^ March 2008) for the West Midlands region of England. These were aggregated into the 3,482 LSOAs that formed small and relatively homogenous units of analysis. We used a negative binomial regression model to investigate the relationship between the number of attenders and distance from the nearest ED, income deprivation, and distance from the nearest minor injuries unit. We also explored interactions between these variables and adjusted for the demographic structure of the neighbourhoods in the study.

### Setting

The study area is the Government Office Region of the West Midlands in which approximately 5.4 million people were resident during the period the study was set [14]. This is a highly heterogeneous region covering large cities such as Birmingham, Stoke-on-Trent, Coventry and Wolverhampton as well as relatively sparsely populated rural areas such as Herefordshire and parts of North Staffordshire.

### Population units

Proximity and deprivation data were based on Lower Level Super Output Areas (LSOAs). The LSOA is a census geography and is commonly used as a unit to explore small area variation in British populations [15]. They are similar in size and function to census tracts used in the U.S [16]. They are neighbourhoods which at the time of the study, had a mean population of 1560 residents. In the region in question, LSOAs are generally small with a median area of 0.45 km^2^ (interquartile range of 0.30 km^2^ to 1.00 km^2^).

### Selection of Participants

Visits captured by the Accident and Emergency Commissioning Data Set (A&CMDS) were included in the study if the attender was resident in the region and attended a “type one” ED. The A&EMDS is a nationally implemented common data set that is captured when someone presents at an ED seeking treatment in the NHS. A type one ED is defined as “a consultant led 24 hour service with full resuscitation facilities and designated accommodation for the reception of accident and emergency patients [17]. This is the typical service model provided by NHS acute hospitals for people needing emergency care, be they walk-ins or ambulance arrivals, and it is attendances at these units that are the subject of this study. MIUs are NHS units which provide a service specifically aimed at people with minor injuries and other less serious conditions. Typically these are provided on the sites of former general hospitals which no longer provide a full range of acute care, but still offer other services such as minor elective surgery or out-patient clinics under the NHS. MIUs are not necessarily led by a consultant in accident and emergency medicine and many do not offer a 24 hour service. Also, they will routinely divert more serious cases (in the event of patients who self-present) to the nearest type one ED.

**Figure 1:**
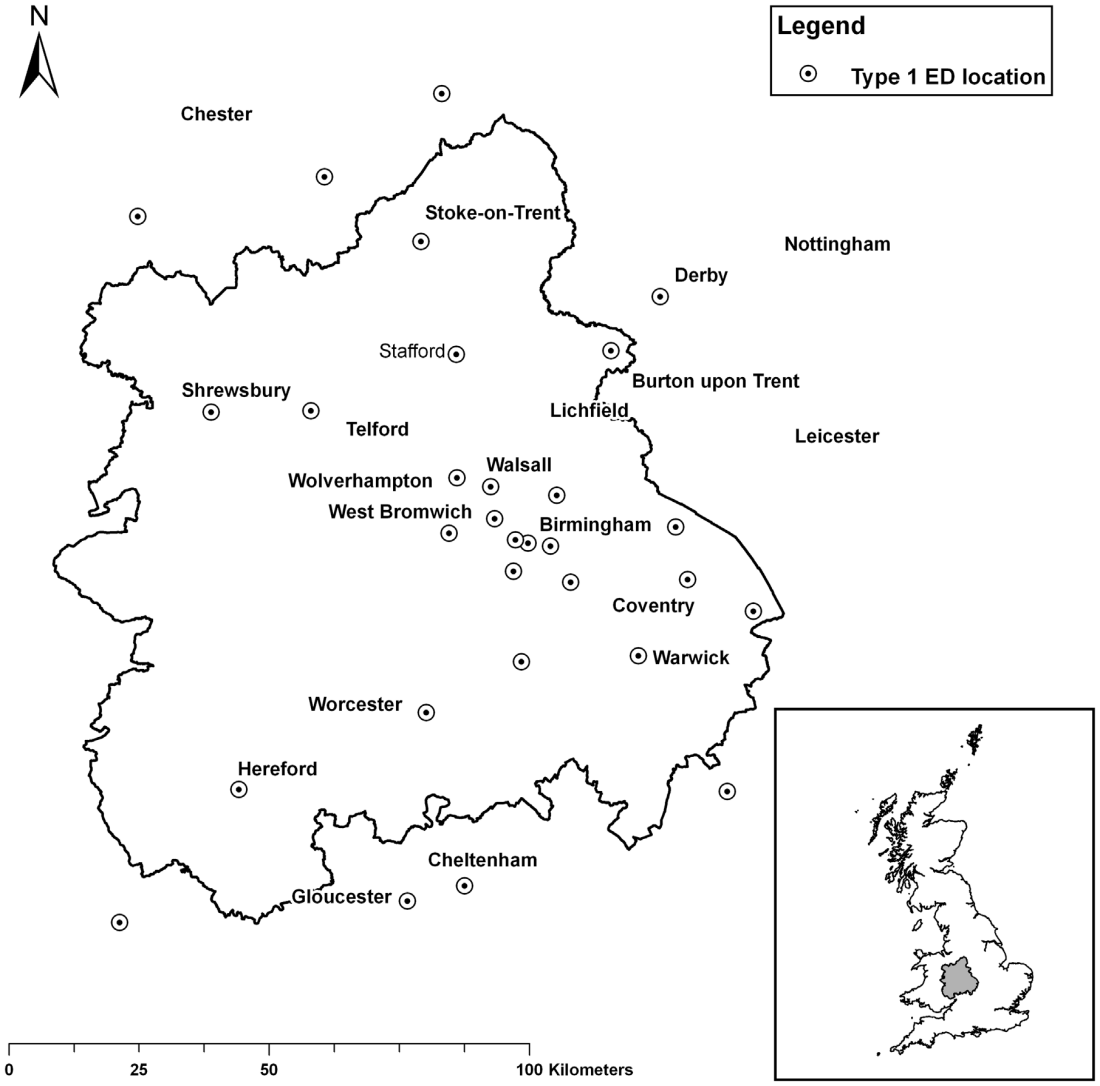
Map of the West Midlands region showing the location of Emergency Departments close to its population in financial year 2007–2008 (inset: the location of the West Midlands region in Great Britain).

At the time of our research, there were twenty-two sites providing type one ED services in the Region, including one specialist children's unit (see figure 1). As we were using a National data set we were also able to capture the (relatively few) ED visits made by residents to EDs outside the region and include them in our analysis.

Attenders were attributed to LSOAs by using their postcode. The postcode is a small unit of geography used to facilitate the delivery of mail. Postcode areas typically cover a few tens of dwellings. We calculated the median size of postcode areas in the West Midlands region as 9,678 m^2^, approximately 2.4 acres. Each one has a mapped polygon determined nationally and digitized for use in geographical information systems (GIS). A national look-up file is maintained by the Office for National Statistics in co-operation with the Royal Mail to attribute postcodes to higher geographies [18]. This is derived by calculating the longitude and latitude of the centre of each polygon and determining in which higher geography, (such as an LSOA) it falls into. This is then used by the NHS to convert patient postcodes captured at or soon after presentation into higher geographies such as LSOAs or local administrative boundaries, in their national data sets. This attribution is done at source and the study used data with the LSOAs already appended to them.

### Methods of Measurement

#### Attendances

ED attendance was determined for the financial year 2007/2008, the most recent year of stable service provision. We had access to later data but chose this year as there were no major reconfigurations or relocations of type 1 EDs in the region during this period and no changes to MIU provision. This was not the case in subsequent years. As we are looking at spatial relationships, we needed these to be constant during the period of observation. We used records of attendance captured by the A&ECDS. Income deprivation was obtained using the Indices of Deprivation 2007 income domain score [19] for each of the LSOAs. This represented the proportion of people in the LSOA who were estimated to live in an income deprived household. Income deprivation is measured as a household having less than 60% of the median national income and/or being in receipt of a number of specified welfare benefits. Rather than using these scores as a continuous variable, which would assume a linear relationship between them and our response variable, we put them into quintiles based upon the rank of deprivation in the region which we treated as categorical variables.

#### Proximity to nearest ED and nearest MIU

The proximity of the LSOAs to the nearest Emergency Department was calculated using Arc GIS software (version 9.3), measuring the centroid of an LSOA which was population weighted centroids of an even smaller geography a Census Output Area. These are the smallest level of geography for which we have population characteristics and aggregated census data. For example, a roughly circular LSOA with a concentration of residents in Census Output Areas at the edge of its northern quadrant and fewer people living elsewhere, would have its distance to hospital estimate pulled closer to that part of the LSOA than the geometric centre. The Ordnance Survey, the UK's national mapping agency, produces digitised transport network data for the country [20]. These data, combined with the GIS software used in the project, were used to calculate the shortest road distances between each of the population weighted centroids described above and the nearest ED and nearest MIU. The researchers obtained the longitude and latitude of all of the EDs in the region using data supplied directly from the NHS Information Centre. We used the “NHS Choices” website [21] to identify and locate all MIUs within a 40 mile buffer of our region. This was deliberately chosen to be larger than necessary to guarantee that the closest unit to any of our LSOAs was captured. We then followed the same methods as used to derive proximity to nearest ED.

### Statistical Analyses

We used two negative binomial models, one for children under fifteen years and one for people aged fifteen and over. For the purposes of this paper we use the term “adult” to refer to people aged fifteen years and over. We chose fifteen years as the cut-off, as this is one of the quinary age group break points in the small area population estimates (provided by the small area population unit at the Office of National Statistics). Attendance counts have a Poisson distribution, with a degree of over-dispersion, so negative binomial regression was chosen as the preferred method. As well as the variables described above we also adjusted for the age composition of the LSOAs by putting the proportion of residents in five year age bands into the model. We also included the proportion of males in each population as a variable and a number of interaction terms. These included the interaction between deprivation and distance and, in the case of adults, the interaction between proportion male and deprivation. We used the overall population of the LSOA as an offset term. All statistical analyses were undertaken using Stata version 11.

## Results

### Characteristics of study subjects

There were a total of 1,413,363 attendances to type 1 EDs which were attributable to residents of the West Midlands. Of these, 288,931 (20.4%) were of children under the age of fifteen years and the remaining 1,124,432 (79.6%) were by those aged 15 years and over.

For the 15 years and over group, 73.9% of all visits were to the nearest provider. For the under 15 years group this was slightly lower at 68.6%, but this was expected, as some activity is directed to the specialist children's ED in Birmingham rather than the nearest ED. Only a very small proportion of visits were made outside of the region of residence; just 1.4% for patients aged 15 and over and 1.2% of visits by children under 15. These percentages did not include the small number of people living at the edges of the region for whom an extra-regional ED was the nearest choice.

### Main results

The model showed that deprivation and distance from hospital had significant effects on attendance. Tables 1 and 2 show the regression correlation coefficients for the two models, one for children under 15 and one for attenders aged 15 and over. In each case the coefficients are expressed as incidence rate ratios (IRR). In both tables the reference group comprises attenders in the least deprived income deprivation quintile.

**Table 1:** Regression co-efficients for a model of child attendance at Emergency Departments.

Variable	IRR	p-value	Lower 95% CI	Upper 95% CI
Deprivation quintile 1	1.000	∼	∼	∼
Deprivation quintile 2	1.344	<0.001	1.199	1.507
Deprivation quintile 3	1.414	<0.001	1.260	1.588
Deprivation quintile 4	1.882	<0.001	1.676	2.115
Deprivation quintile 5	2.198	<0.001	1.904	2.537
Distance to nearest ED (km)	0.978	<0.001	0.974	0.983
Deprivation quintile 2 * distance to ED	0.986	<0.001	0.980	0.992
Deprivation quintile 3 * distance to ED	0.984	<0.001	0.978	0.990
Deprivation quintile 4 * distance to ED	0.972	<0.001	0.965	0.979
Deprivation quintile 5 * distance to ED	0.962	<0.001	0.952	0.972
Distance to nearest MIU (km)	1.049	<0.001	1.044	1.054
Deprivation quintile 2 * distance to MIU	0.996	0.028	0.992	1.000
Deprivation quintile 3 * distance to MIU	0.997	0.103	0.993	1.001
Deprivation quintile 4 * distance to MIU	0.992	<0.001	0.988	0.996
Deprivation quintile 5 * distance to MIU	0.994	0.025	0.989	0.999
Distance to nearest MIU (km)?2	0.999	<0.001	0.999	0.999
Proportion population aged 5–9	1.288	<0.001	1.150	1.443
Proportion population aged 5–9?2	0.996	<0.001	0.994	0.998
Proportion population aged 10–14	0.923	<0.001	0.892	0.955
Proportion population aged 10–14?2	1.001	<0.001	1.000	1.001

**Table 2:** Regression co-efficients for a model of adult attendance at Emergency Departments.

Variable	IRR	p-value	Lower 95% CI	Upper 95% CI
Deprivation quintile 1	1.000	∼	∼	∼
Deprivation quintile 2	1.346	<0.001	1.228	1.475
Deprivation quintile 3	1.481	<0.001	1.348	1.626
Deprivation quintile 4	1.865	<0.001	1.697	2.049
Deprivation quintile 5	2.259	<0.001	2.006	2.545
Distance to nearest ED (km)	0.985	<0.001	0.981	0.988
Deprivation quintile 2 * distance to ED	0.988	<0.001	0.984	0.993
Deprivation quintile 3 * distance to ED	0.987	<0.001	0.982	0.992
Deprivation quintile 4 * distance to ED	0.980	<0.001	0.975	0.985
Deprivation quintile 5 * distance to ED	0.969	<0.001	0.962	0.977
Distance to nearest MIU (km)	1.042	<0.001	1.038	1.046
Deprivation quintile 2 * distance to MIU	0.996	0.014	0.993	0.999
Deprivation quintile 3 * distance to MIU	0.996	0.010	0.993	0.999
Deprivation quintile 4 * distance to MIU	0.994	0.001	0.991	0.997
Deprivation quintile 5 * distance to MIU	0.996	0.050	0.991	1.000
Distance to nearest MIU (km)?2	0.999	<0.001	0.999	0.999
Proportion population male	1.007	0.003	1.002	1.011
Proportion population aged 15–19	0.996	0.346	0.987	1.005
Proportion population aged 20–24	0.984	0.039	0.969	0.999
Proportion population aged 25–29	1.053	<0.001	1.025	1.081
Proportion population aged 30–34	0.965	0.001	0.945	0.986
Proportion population aged 35–39	1.001	0.911	0.979	1.024
Proportion population aged 40–44	1.016	0.170	0.993	1.038
Proportion population aged 50–54	0.976	0.053	0.952	1.000
Proportion population aged 55–59	0.950	<0.001	0.928	0.974
Proportion population aged 60–64	1.032	0.002	1.011	1.053
Proportion population aged 65–69	1.014	0.296	0.988	1.039
proportion population aged 70–74	0.962	0.003	0.937	0.987
Proportion population aged 75–79	1.005	0.709	0.979	1.032
Proportion population aged 80–84	1.038	0.009	1.009	1.068
Proportion population aged > = 85	0.978	0.002	0.965	0.992

For adult attendances, our results show that, having adjusted for the other variables, there is a significant reduction in attendance associated with increasing distance from an ED, of about 1.5% per kilometre in the reference group. This is a large effect size given the variation that exists in distance from EDs across the region, especially in rural areas. We also see a significant effect of deprivation. Using the least deprived quintile as a reference group, each subsequent quintile of deprivation is associated with higher attendances than the last, with attendance in the most deprived quintile being more than twice as large as that in the least deprived adjusting for the other variables (IRR 2.26, 2.01–2.55, *p*<0.001).

Children's attendances decreased with distance from ED more than those of adults, with one kilometre of extra distance being associated with a fall of 2.2% in attendance in the reference group; although the effect of deprivation was broadly similar.

**Figure 2:**
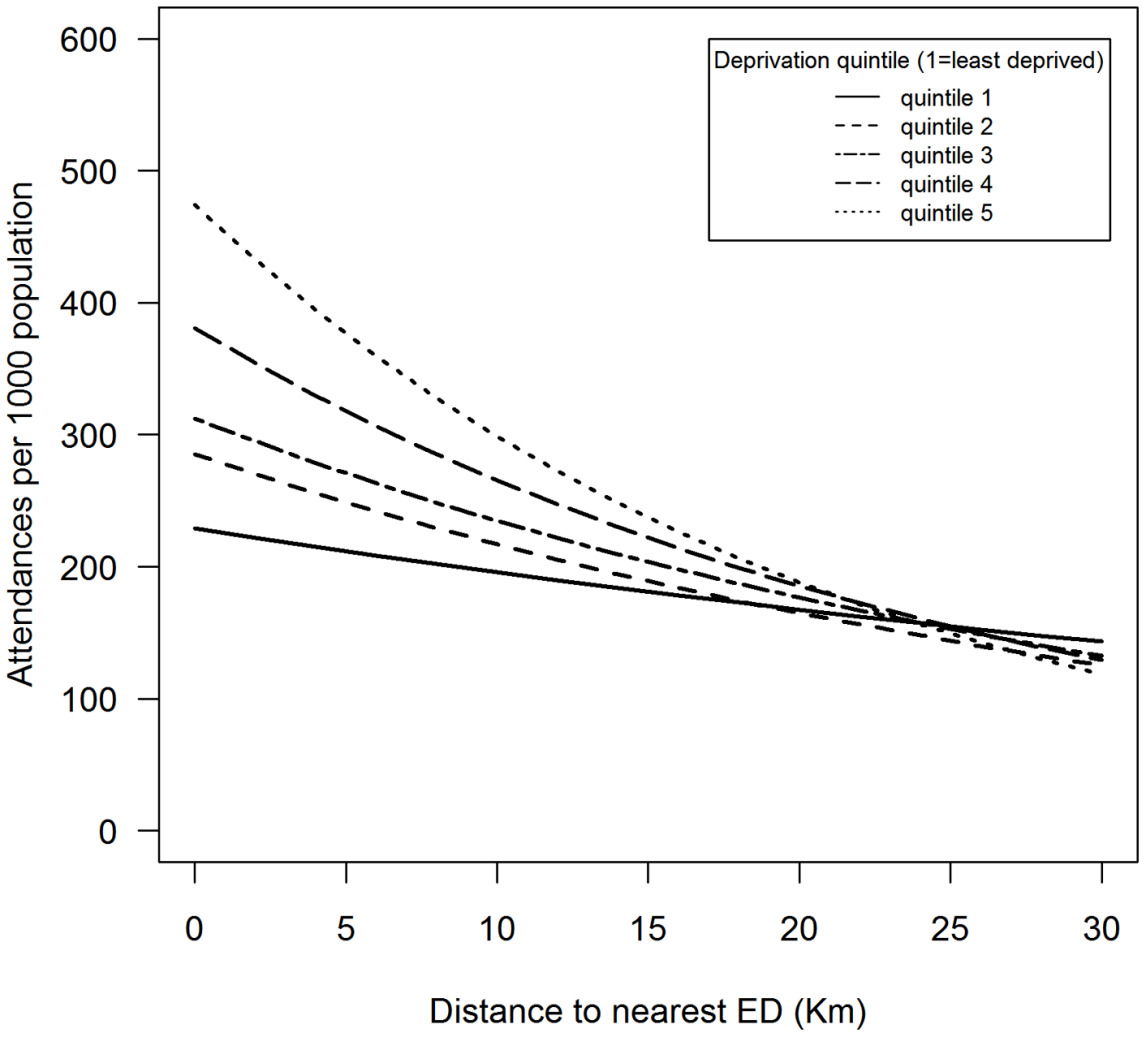
Modelled attendance change with distance at various levels of neighbourhood income deprivation, attenders aged > = 15.

**Figure 3:**
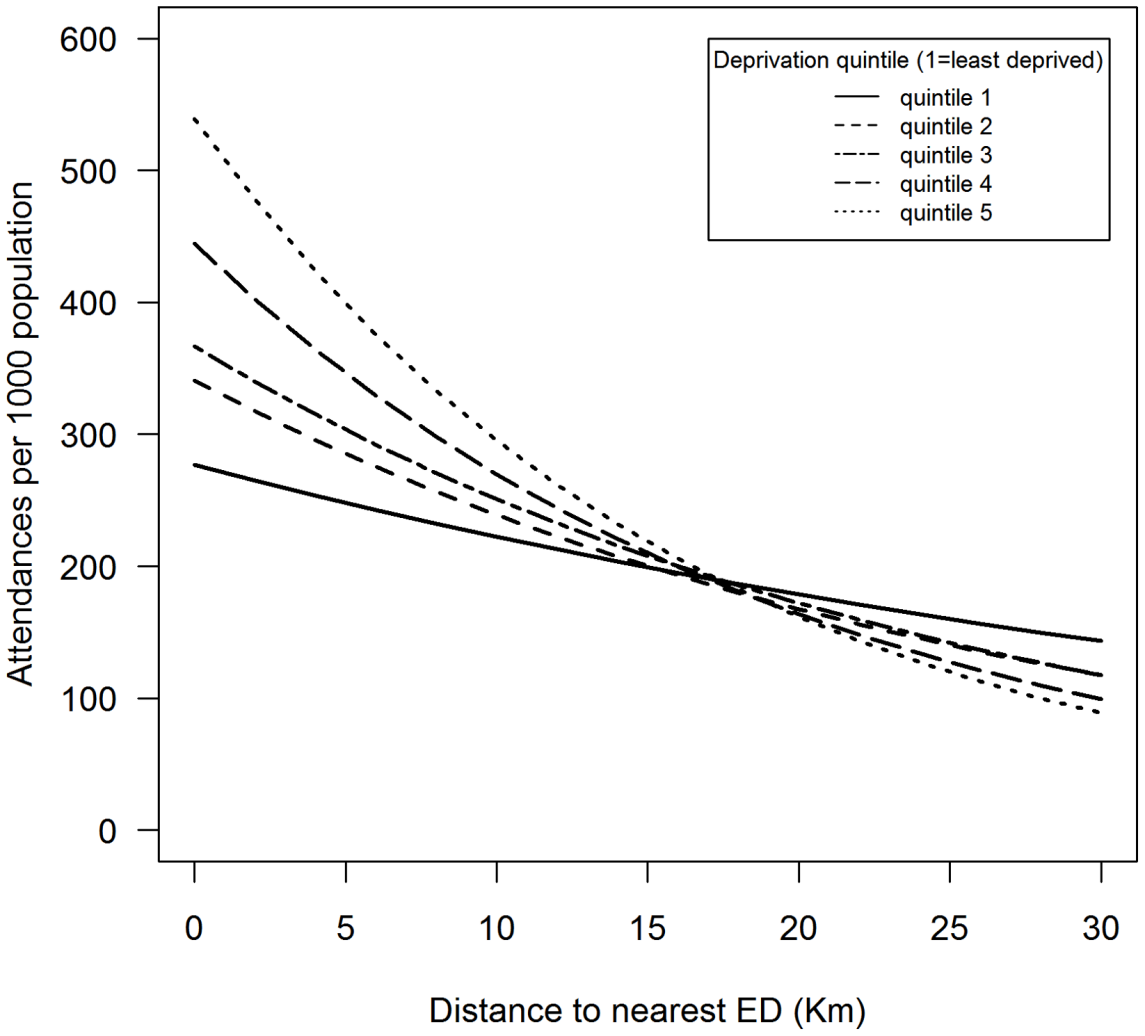
Modelled attendance change with distance at various levels of neighbourhood income deprivation, attenders aged <15.

The interaction between deprivation and distance was highly significant in both models at all levels of deprivation (*p* = <0.001). Attendance in deprived neighbourhoods reduces with distance to a much greater degree than attendance in less deprived ones. This is the case for both adults and children. From the IRRs alone this is not straightforward to interpret. To illustrate the effect, two figures have been provided, Figure 2 and Figure 3. These plots show the predicted effect on attendance with distance at the five deprivation quintiles, holding the distance to MIU constant at its median, and the other variables at their mean. This shows the modeled net effect of distance, increasing at higher levels of deprivation. In both adults and children we see the predicted levels of attendance being higher at each level of deprivation, but then we see the decrease in attendance with distance being much greater in more deprived populations. This is more marked in children than in adults, particularly in the attendance of children at the level of the most deprived quintile.

There is a significant positive association between distance to an MIU and attendance at a type one ED in both children and adults. This predicts that for each additional kilometre away from an MIU, type one ED use will increase by 4.2% for adults (3.8%–4.6%, p<0.001) and 4.9% for children (4.4%–5.4%, p<0.001). Again we see an interaction effect with deprivation. This suggests that the extent to which people in communities with different levels of deprivation use MIUs, differs with respect to distance.

## Discussion

We have found that ED use is significantly associated with both deprivation and distance and that attendance in deprived neighbourhoods reduces more with distance from hospital than in less deprived ones. The effect is more marked for children's attendances than those of adults.

Previous studies of distance effects have relied on more limited study areas and are not consistent. Both Hull et al [1] and Walsh [3] found significant associations between attendance and both deprivation and distance. However, both were based on more geographically limited populations than the one in this study, and were limited to adults registered with a GP. The latter study was also limited to patients discharged home on the day of discharge. Other studies investigating the effects of distance and deprivation on attendance have reached different conclusions. McKee et al [5] found that attendance in electoral wards in a rural area of Northern Ireland declined significantly with distance from hospital, but suggested that deprivation did not increase the explanatory power of the regression model they used. Conversely Carlisle et al, [2] in a study set in Nottingham in England, suggested that the apparent effect of distance was nearly wholly explained by deprivation and that distance net of deprivation, had a limited effect. In the Nottingham study subjects were limited to patients registered with a GP who attended out of hours.

The finding regarding the effect of MIU proximity is interesting. It appears that being further away from an MIU is associated with a much greater propensity to attend a type 1 ED however MIUs typically serve more rural areas so those few populations who happen to live in close proximity to them are not typical. There is very little literature on the impact of MIUs in reducing type 1 ED use, evaluations having being more focused on things like patient acceptability and patient satisfaction. However given the proliferation of them, further research on this topic would be timely and useful.

### Advantages and limitations of this study

This study is of a large, heterogeneous and geographically contiguous population. We used a data set which is mandated in hospitals nationally and there are no non-state provided EDs in the English healthcare system, so our capture of attendance is near complete. The measure of deprivation we used is considered generally robust. As LSOAs are small units designed to facilitate the statistical analysis of the national population, they usefully have a high level of homogeneity. The distance variable was based upon actual road distances rather than Euclidian distances that may have been subject to error, particularly in rural areas. Although the distance variable based upon actual road distances, it used distance from population weighted centroids as a proxy for distance to hospital for all households in a neighbourhood and so has a degree of error, likely to be larger in larger rural LSOAs. We used projected populations, some time from the 2001 census, which need a degree of caution, but represent the best estimate available.

We have not adjusted for case severity. Concerns about the effect of adjusting for case mix, especially using administrative data have been raised in the literature [22], [23], [24]. In addition we had concerns about the quality of capture of specific items we might have used to carry out such an adjustment. The A&ECDS captures up to two presenting complaints using a simple numeric code (although some units use the International Classification of Diseases version 10, to which these codes do not map). The degree to which these data are well captured, is highly variable [25]. To verify this we undertook a sub-analysis of the coding of the reason for attendance and the proportions of visits resulting in an admission. A high degree of variability in these data points would support our decision to avoid case mix adjustment. In this sample the proportion of visits with a missing or invalid diagnosis code in the primary diagnosis on arrival varied between This varied from zero to 65.1%. The proportion of visits with a ‘diagnosis unclassifiable’ flag varied from zero to 45.7% and the admission rate varied between 14.6% to 36.4% between hospitals.

### Interpretation

The reasons why deprived populations and populations close to EDs, use them more often does warrant further study. It is evident that deprived populations have higher incidences of serious illness, resulting in more non-negotiable needs for emergency care. However it is also possible that a higher proportion of attendances in these populations are for less urgent needs, in which case the cost of time and transport may dissuade presentations from further away especially if these problems could normally be addressed through primary care. A Canadian study showed that there was a significant decay with distance for less urgent cases, but not for more urgent ones [6].

In seeking treatment for minor ailments, patients can consult a GP (which is also free of charge in the UK) although getting appointments at short notice or out of normal working hours can be problematic [26]. There is evidence that there are more actual or perceived barriers to accessing good quality primary care in deprived areas. Firstly there are likely to be more people (who are not registered with a GP (for example transients and recent migrants) and who may be unaware that they can access primary care as a temporary resident. For example, in a study of a sample of Eastern European ED attenders (carried out in the same region in which our study was set) it was found that 43% were unregistered, compared to a rate of 7.4% observed in all attenders [27]. There is also evidence that people of lower socio-economic status are less likely to be satisfied with the quality of primary care available to them [28] and that more deprived people have different expectations of primary care including a lower willingness to travel to access it [29]. Campbell [4] explored this issue and detected a correlation between ED use and heightened dissatisfaction with GP services, but not with other variables such as appointment system functionality. Research using the NHS's Quality and Outcome Framework, which measures aspects of GP practice quality, have found associations between lower quality scores and the deprivation of the population served [30].

More deprived service users or inner-city dwellers who live close to hospitals may access care differently. Studies of ED attenders in Denmark found that migrants were significantly more likely to present at EDs than the indigenous population [31]. However, even if recent migrants do use EDs differently, their relatively small numbers could probably not explain the extreme variation seen in the use of EDs, even at small area level.

The more marked distance effect in children may be due to there being a higher proportion of less serious health needs in this population. The incidence of life threatening medical emergencies is very small in child populations compared with that in adults where events such as strokes and heart attacks, particularly in older people, are more common. A study of injuries in children in Wales showed that there was a significant decrease with distance in injury presentations generally, but in fractures the distance effect was not significant suggesting that more discretionary attendances will be more sensitive to distance [32].

The finding that proximity to MIUs did influence the number of ED visits was expected, given that we would expect minor injury to be a common reason to attend a type one ED. However, we need to be cautious in the interpretation of these results and especially the apparently large effect size. The reference for the intercept of the modelled attendance assumes no distance from an MIU. The populations who live close to MIUs are predominantly rural, less heterogeneous than urban ones, and tend to use EDs less. Whilst we have adjusted for demography and socio-economics, there still may be some unmodelled variation which disproportionately affects the way in which these people use services and which may cause a gradient of increasing use with distance from MIUs. Further research is needed on MIUs however, as they have been set up in large numbers at some cost to the health service, but relatively little is known about how or indeed if, they divert demand from other services.

## Conclusions

There are two important policy implications of our findings. Firstly, a number of reconfigurations of hospital service are underway in England. Some of these will result in a step-change in the distance to provider in a densely populated area. These models suggest that this may cause unexpected step-changes in local demand patterns for emergency care, independent of the demographic, socio-economic and even epidemiological conditions extant in that area.

Secondly, if a disproportionate amount of demand for ED services from deprived areas close to hospitals is indeed better dealt with in a primary care setting, it may be possible to modify health seeking behaviour in these areas with a social marketing approach. Campaigns such as “choose well” [33] have been organised by the NHS to encourage more responsible use of EDs. A model such as the one we have developed here could be useful in suggesting locations for more targeted use of such initiatives.
